# Supplementary material for: Autophagy disruption and mitochondrial stress precede photoreceptor necroptosis in multiple mouse models of inherited retinal disorders
Source: Nat Commun. 2025 Apr 29;16:4024. doi: 10.1038/s41467-025-59165-8 (PMC12041483; doi:10.1038/s41467-025-59165-8)
Supplement: Supplementary file 1 — Supplementary Information [file 41467_2025_59165_MOESM1_ESM.pdf]

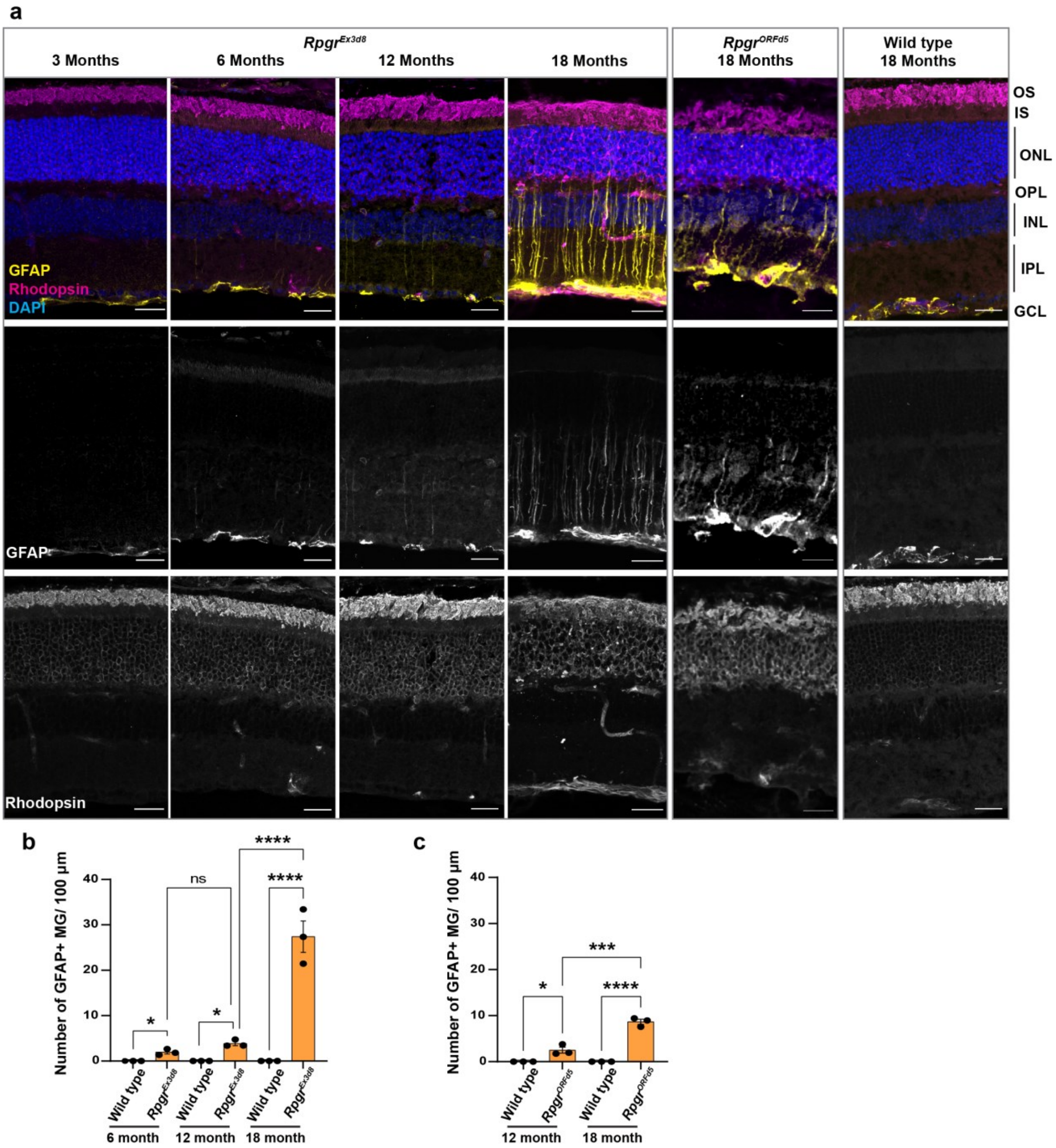

Supplementary figure 1: *Rpgr* mutants exhibit progressive retinal stress and rhodopsin mis-localisation.

(**a**) Reactive gliosis, as evidenced by increased GFAP immunolabelling throughout the radial length of Müller glial cells in the outer nuclear layer, increases in *Rpgr*^{Ex3d8} and *Rpgr*^{ORFd5} mutant retinas over time from 6 months of age (prior to significant ONL thinning at 18 months). Rhodopsin accumulates in inner segments and cell bodies at later time points (scale bars = 25 µm). OS = photoreceptor outer segments, IS = photoreceptor inner segments, ONL = outer nuclear layer, OPL = Outer plexiform layer, INL = inner nuclear layer, IPL = inner plexiform layer, GCL = ganglion cell layer. (**b**, **c**) Quantification of GFAP+ Müller glia (MG) in *Rpgr*^{Ex3d8} at 6, 12 and 18 months (**b**) and *Rpgr*^{ORFd5} at 12 and 18 months (**c**). Bars indicate mean; n = 3 mice per genotype at each time point; error bars show SEM. **b** * p = 0.038 (6 months), p = 0.014 (12 months), **** p = 0.0001 (18 months); by unpaired t test. **** p = 3.36E-05 (12 versus 18 months) by 2-way ANOVA.

c * p = 0.014 (12 months), **** p = 0.0001 (18 months) by unpaired t test; *** p = 0.002 (12 versus 18 months) by 2-way ANOVA.

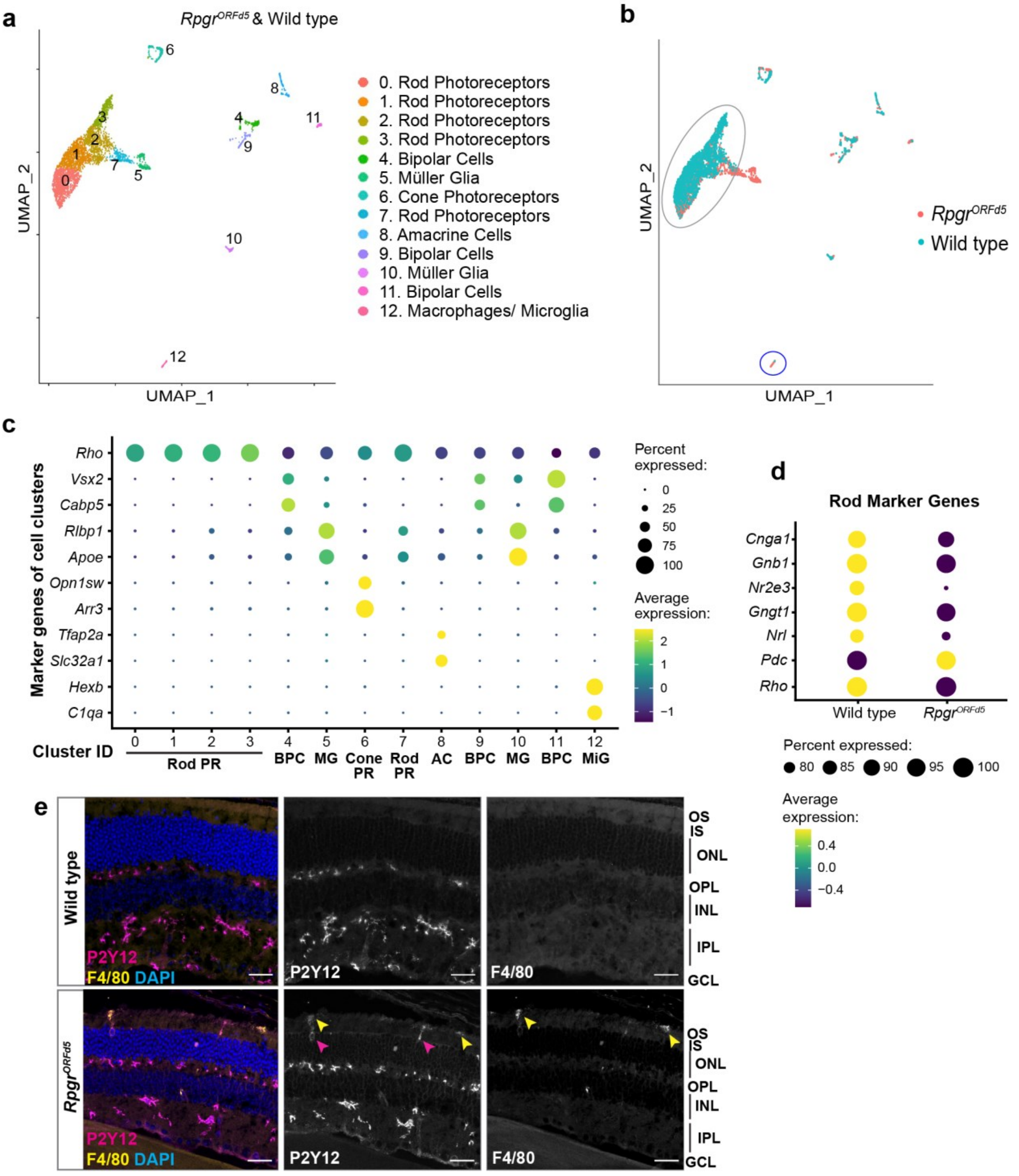

Supplementary figure 2: Single-cell transcriptomics identifies novel cell populations in *Rpgr* ^{ORFd5} mutant retina.

(**a**) UMAP plot showing clusters corresponding to photoreceptors and other retinal cell types in *Rpgr* ^{ORFd5} retinas at 18 months (combined data from mutants and wild-type littermate controls, coloured by cluster identity, numbered by cluster size). (**b**) *Rpgr* ^{ORFd5} mutant cells in salmon overlaid with wild-type cells in cyan. Rod photoreceptor clusters are circled in grey, macrophages (mainly

Rpgr^{ORFd5} cells) circled in blue. (**c**) Dot plot showing expression of retinal cell type-specific marker genes in each cluster (combined mutant and wild-type data, indicating cell type identity of each cluster (PR = photoreceptors, BPC = bipolar cells, MG = Müller glia, AC = amacrine cells, MiG = microglia/ macrophages). (**d**) Rod photoreceptor marker genes are downregulated in *Rpgr*^{ORFd5} mutant rod PR cells compared to wild type. (**e**) Macrophage cells (F4/80^{high}; P2Y12^{low}, yellow arrowheads) and microglia (F4/80^{low}; P2Y12^{high}, magenta arrowheads) are present in outer retinal layers in *Rpgr*^{ORFd5} mutants but not wild-type littermate controls at 18 months (scale bar = 25 µm). OS = photoreceptor outer segments, IS = photoreceptor inner segments, ONL = outer nuclear layer, OPL = Outer plexiform layer, INL = inner nuclear layer, IPL = inner plexiform layer, GCL = ganglion cell layer.

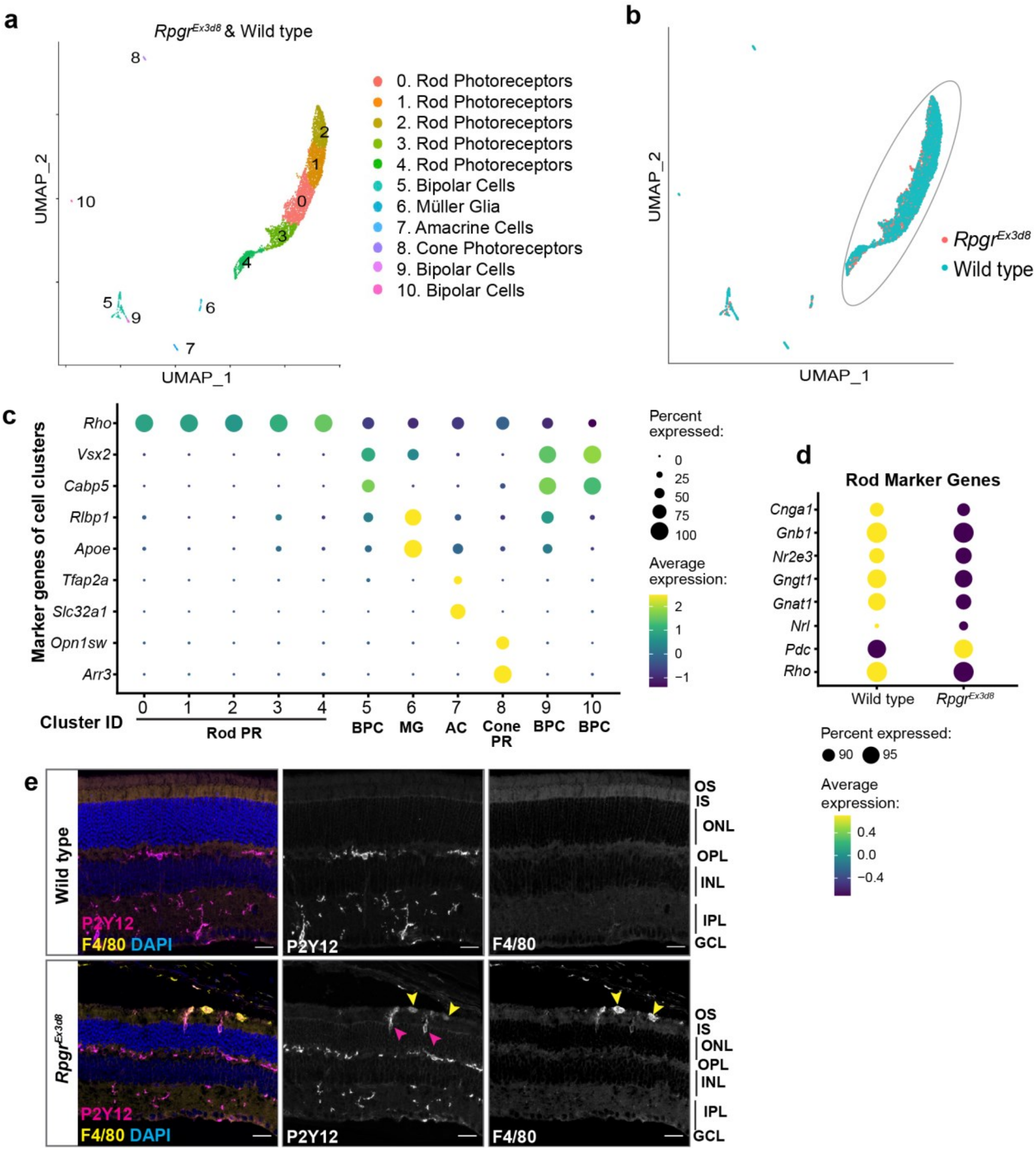

Supplementary figure 3: Single-cell transcriptomics identifies novel cell populations in *Rpgr^{Ex3d8}* mutant retina.

(**a**) UMAP plot showing clusters corresponding to photoreceptors and other retinal cell types in *Rpgr^{Ex3d8}* retinas at 18 months (combined data from mutants and wild-type littermate controls, coloured by cluster identity). (**b**) *Rpgr^{Ex3d8}* mutant cells in salmon overlaid with wild-type cells in cyan. Rod photoreceptor clusters are circled in grey. (**c**) Dot plot showing expression of retinal cell type specific marker genes in each cluster (combined mutant and wild-type data), indicating cell type identity of each cluster (PR = photoreceptors, BPC = bipolar cells, MG = Müller glia, AC = amacrine cells). (**d**) Rod photoreceptor marker genes are downregulated in *Rpgr^{Ex3d8}* mutant rod PR cells compared to wild type. (**e**) Although macrophage cells were not present in the scRNAseq data for this mutant, Macrophages (F4/80^{high}; P2Y12^{low}, yellow arrowheads) and microglia (F4/80^{low}; P2Y12^{high}, magenta arrowheads) are present in outer retinal layers in *Rpgr^{Ex3d8}* mutants but not wild-

type littermate controls at 18 months (Scale bar = 25 µm). OS = photoreceptor outer segments, IS = photoreceptor inner segments, ONL = outer nuclear layer, OPL = Outer plexiform layer, INL = inner nuclear layer, IPL = inner plexiform layer, GCL = ganglion cell layer.

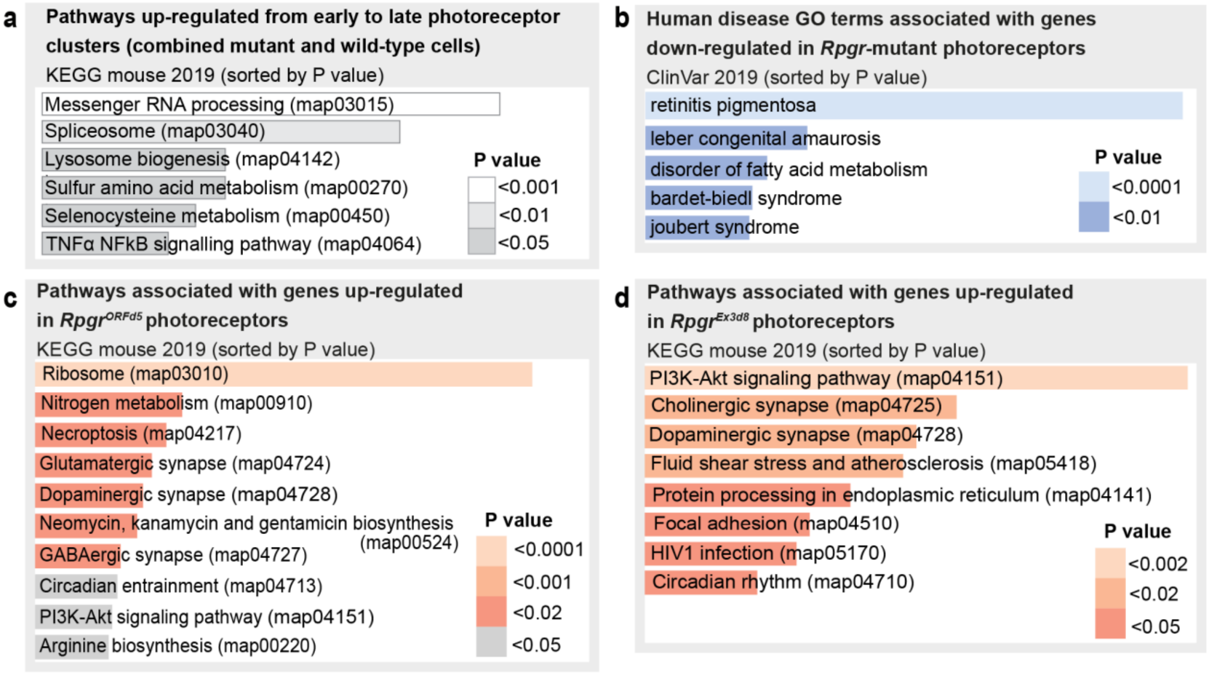

Supplementary figure 4: Cell stress pathways and PI3K-AKT signalling are up-regulated in degenerating *Rpgr* mutant photoreceptors.

GO term and KEGG pathway enrichment analyses performed using Enrichr[1-3]. Bar length represents number of differentially expressed genes associated with each GO term/ KEGG pathway. (**a**) Pathways upregulated in rod photoreceptor subclusters along the disease trajectory: 'early' (less degenerated) to 'late' (more degenerated) in combined wild type and mutant cells. (**b**) Human disease GO terms associated with down-regulated genes identified from mutant photoreceptors compared to wild-type littermate controls. (**c, d**) Pathways up-regulated in *Rpgr*^{ORFd5} (**c**) and *Rpgr*^{Ex3d8} (**d**) photoreceptors compared to wild-type littermate controls.

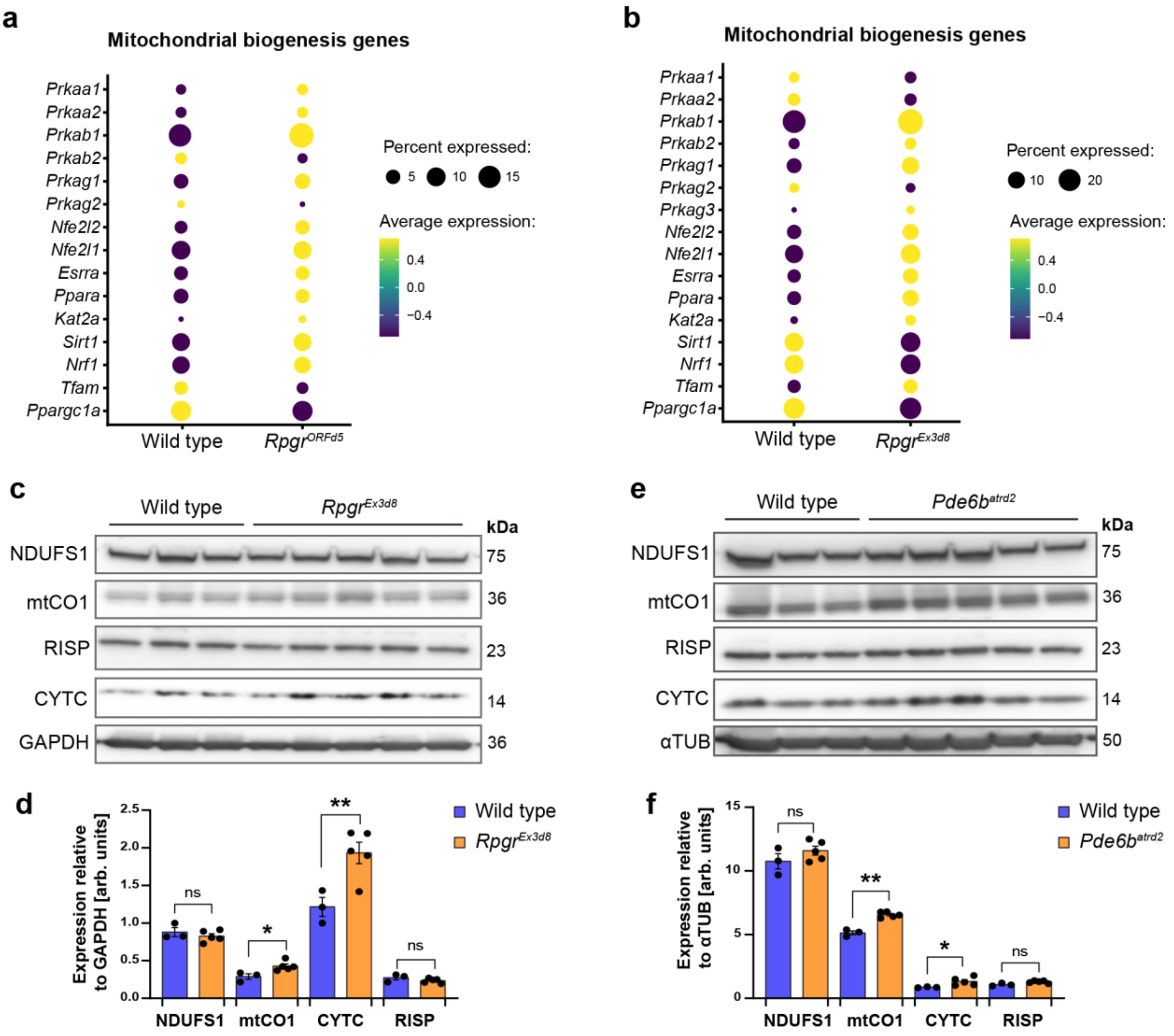

Supplementary figure 5: Mitochondrial biogenesis and oxidative phosphorylation are up-regulated in *Rpgr* and *Pde6b*^{atrd2} mutants

(**a, b**) Dot plots showing expression level of mitochondrial biogenesis genes in *Rpgr*^{ORFd5} (**a**) and *Rpgr*^{Ex3d8} (**b**) mutant versus wild type rod photoreceptors in scRNAseq experiments. (**c**) Expression of oxidative phosphorylation proteins in *Rpgr*^{Ex3d8} and wild type retina lysates at 12 months, quantified in (**d**) (n = 3 wild type mice, n = 5 mutant mice; bars show mean expression level; error bars show SEM; * p = 0.036, ** p = 0.0099 by unpaired t test). (**e**) Expression of oxidative phosphorylation proteins in *Pde6b*^{atrd2} and wild type retina lysates at P21, quantified in (**f**) (n = 3 wild type mice, n = 5 mutant mice; bars show mean expression level; error bars show SEM; * p = 0.033, ** p = 0.002, by unpaired t test).

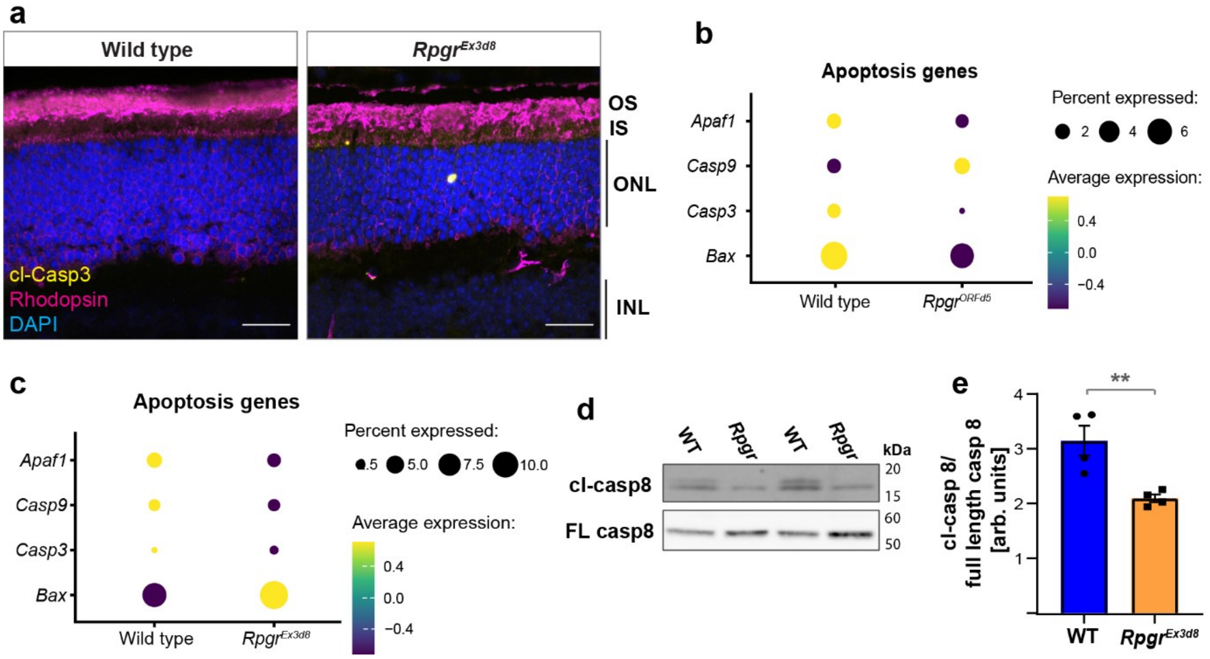

Supplementary figure 6: Apoptosis may not be the major cell death mechanism in *Rpgr* mutants.

(**a**) Cleaved caspase-3 positive photoreceptors are present in *Rpgr*^{Ex3d8} mutant retinas at 18 months compared to wild type littermate controls, scale bar = 25µm. (**b, c**) Expression of apoptosis genes in *Rpgr*^{ORFd5} and *Rpgr*^{Ex3d8} mutants. (**d, e**) Immunoblot shows reduced cleaved caspase-8 relative to full-length caspase-8 in *Rpgr*^{Ex3d8} mutant retina lysates (n = 4 animals per experimental group; error bars show SEM; ** p = 0.0065 by unpaired t test).

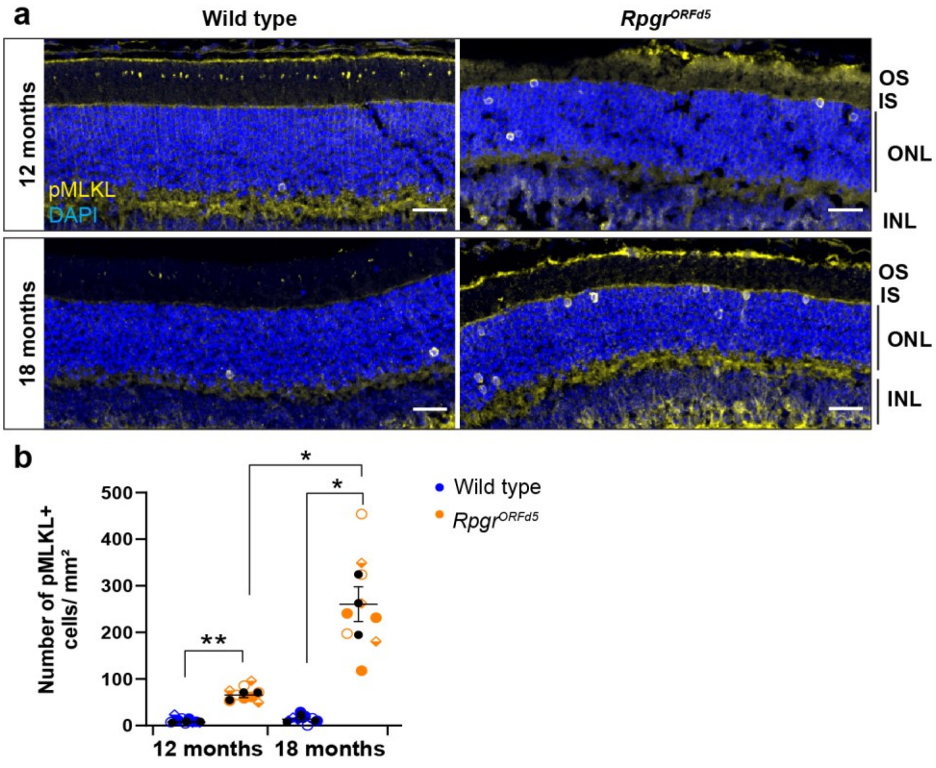

Supplementary figure 7: Progressive increase in photoreceptor necroptosis in *Rpgr*^{ORFd5} mutants.

(**a**) Increased pMLKL positive photoreceptors in *Rpgr*^{ORFd5} mutants at 12 and 18 months compared to wild type littermate controls (scale bar = 25 µm). (**b**) Symbols indicate images from individual mice (N = 3 animals per condition, means for each animal are overlaid in black circles; bars show mean; error bars show SEM; for each time point means for individual mutant and wild type animals were compared by unpaired t tests with Welch's correction * p = 0.02 (18 months), ** p = 0.007; Comparisons between time points were analysed by 2-way ANOVA, * p = 0.033 (12 vs 18 months)).

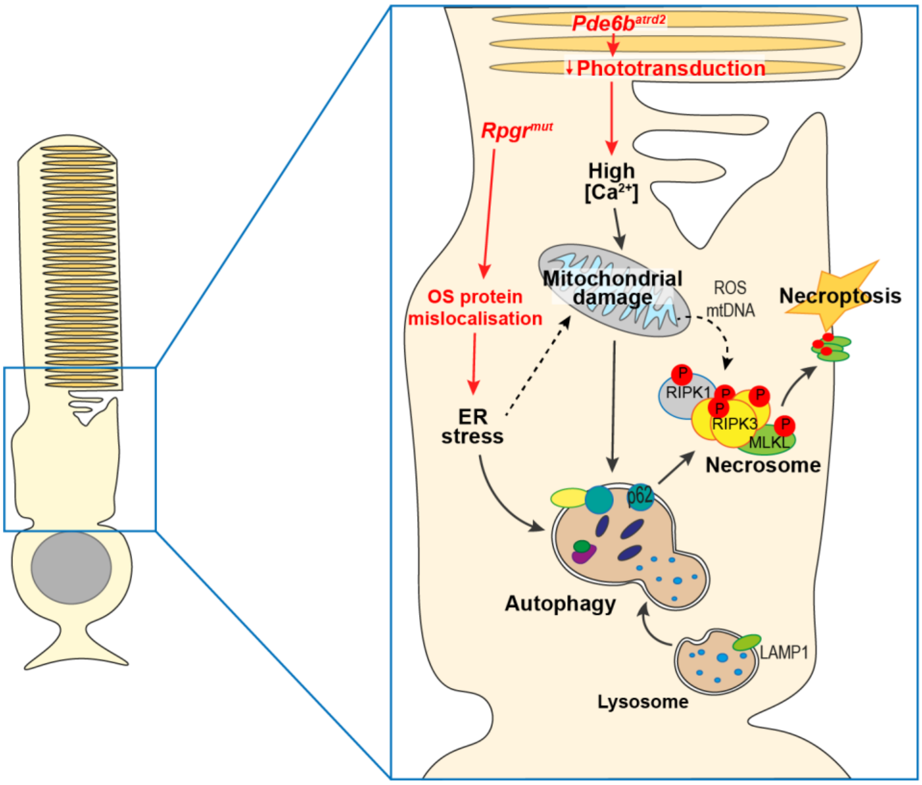

Supplementary figure 8. Different mechanisms of photoreceptor damage in *Rpgr* and *Pde6b*^{atrd2} mutants descend on a common cell death pathway.

Phototransduction is impaired in *Pde6b*^{atrd2} mutants, possibly leading to raised intracellular calcium and mitochondrial damage. Outer segment maintenance is impaired in *Rpgr* mutants, leading to mislocalisation of OS proteins, increased endoplasmic reticulum (ER) stress and mitochondrial damage. Autophagy, to remove damaged mitochondria, increases in early disease. Necroptosis is facilitated by increased autophagy (via interaction of p62 with the necrosome), and release of reactive oxygen species (ROS) and mitochondrial DNA (mtDNA) from damaged mitochondria, facilitating necrosome formation.

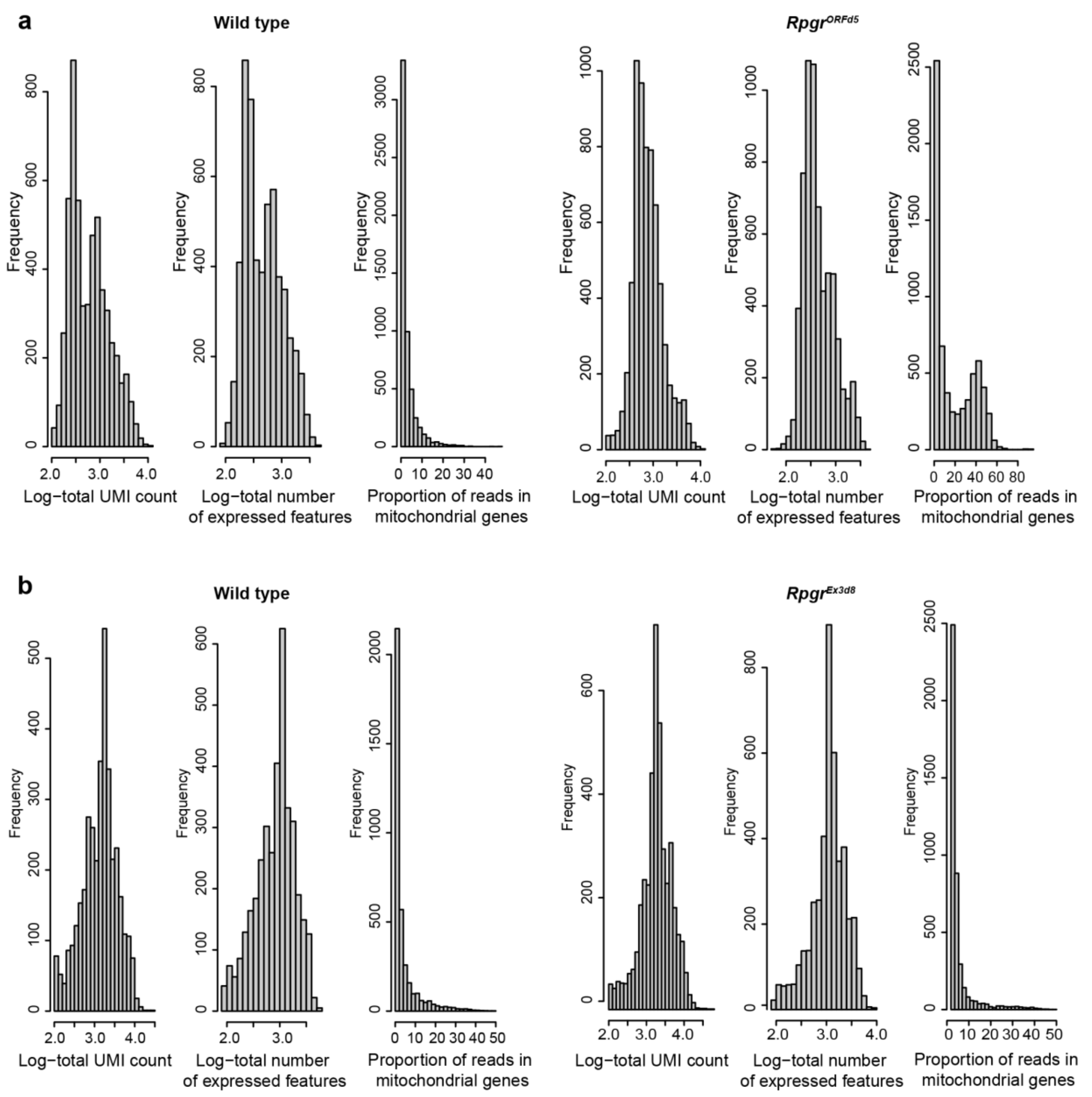

Supplementary figure 9: Cell-level quality control performed by Scater.

Diagnostic plots for cell-level QC in scRNAseq data for *Rpgr*^{ORFd5} (**a**) and *Rpgr*^{Ex3d8} (**b**) retinas investigating the distribution of total number of counts for the cell (Log-total UMI count), the total number of features for the cell (Log-total number of expressed features) and the percentage of all counts for the cell that come from mitochondrial genes (Proportion of reads in mitochondrial genes).

Classification	Cluster number WT + ***Rpgr***^{ORFd5}	Cluster number WT + ***Rpgr***^{Ex3d8}	Marker Genes	Adjusted P value WT + ***Rpgr***^{ORFd5}	Adjusted P value WT + ***Rpgr***^{Ex3d8}
Photoreceptors (rods)	3	4	*Rho*	4.87E-186	5.55E-201
			Cpe	9.34E-143	3.97E-292
			Pde6g	1.69E-184	5.86E-239
			Tma7	2.66E-174	4.85E-289
			Gngt1	1.80E-179	3.41E-293
			Rcvrn	1.31E-147	1.18E-300
			Vtn	2.35E-139	8.25E-208
			Ndufa4	4.08E-111	3.46E-303
	2	3	*Gnat1*	1.07E-26	4.41E-32
			Rcvrn	1.28E-31	3.33E-44
			Pdc	8.51E-33	2.70E-46
	-	0	*Ypel2*	N/A	2.16E-13
	1	1	*Tmem108*	2.16E-26	6.08E-22
			Sgk1	4.73E-38	2.66E-18
			Marchf1	1.46E-23	2.27E-25
			Pex5l	1.32E-11	8.62E-19
			Rsrp1	8.57E-28	6.31E-25
			Rabgef1	7.00E-06	1.68E-07
	0	2	*Rpgrip1*	4.28E-109	3.32E-60
			Dmd	4.66E-53	9.26E-30
			Atp11b	2.56E-12	3.36E-03
			Slc4a7	8.34E-21	1.13E-11
			Cspp1	1.01E-12	4.09E-03
			Cep164	6.19E-25	1.07E-03
			Grk1	7.78E-17	3.81E-03
Photoreceptors (cones)	6	8	*Opn1sw*	9.61E-207	3.50E-161
			Pde6h	8.92E-186	4.80E-183
			Arr3	3.92E-139	4.13E-94
			Opn1mw	9.16E-134	2.90E-98
			Gnat2	4.41E-174	4.56E-275
			Gngt2	6.38E-263	7.25E-205
			Pde6c	2.81E-178	2.37E-50
			Gnb3	2.00E-172	3.37E-46
Bipolar cells 1	4	5	*Scg2*	1.03E-199	4.25E-290
			Neurod4	7.07E-266	3.80E-134
			Gng13	1.09E-302	7.29E-132
			Cabp5	9.27E-262	4.51E-140
			Nrxn3	2.61E-251	4.77E-201
			Isl1	2.90E-260	7.92E-104
			Gnao1	1.37E-199	3.80E-134
			Prox1	1.37E-237	1.13E-99
Bipolar cells 2	9	9	*Gsg1*	1.55E-227	9.80E-83
			Pcp4	1.01E-68	7.10E-182
			Grik1	1.61E-162	1.46E-94
			Rskr	2.11E-158	2.08E-90
			Lhx4	5.27E-135	1.04E-184
			Otor	1.37E-205	2.55E-175
Bipolar cells 3	11	10	*Pcp2*	2.57E-179	1.72E-128

			Trpm1	7.07E-153	3.61E-122
			Gng13	1.17E-118	1.36E-93
			Pcp4	4.54E-113	2.41E-121
			Chgb	1.87E-78	5.00E-49
			Car8	1.60E-283	3.83E-224
			Calm1	1.83E-43	1.53E-23
			Isl1	1.78E-190	1.15E-163
Müller Glia	10	6	*Apoe*	1.93E-100	2.88E-163
			Clu	4.27E-132	2.24E-194
			Dkk3	3.50E-293	3.82E-89
			Glul	3.61E-64	1.22E-78
			Trpm3	2.88E-192	4.28E-205
			Rlbp1	1.13E-75	1.05E-118
Amacrine cells	8	7	*Snhg11*	7.73E-221	9.06E-76
			Cartpt	1.12E-107	1.08E-249
			Tkt	5.43E-138	3.76E-109
			Celf4	4.80E-108	1.28E-58
			Gad1	3.01E-136	4.23E-157
			Lamp5	6.80E-291	3.18E-139
			Elavl3	2.63E-203	1.54E-116
Microglia/ macrophages	12	-	*Ctss*	2.04E-52	N/A
			C1qa	6.60E-124	
			C1qb	2.03E-56	
			Hexb	8.07E-126	
			Ctsd	4.63E-41	
			Tyrobp	1.56E-272	
			Cx3cr1	2.22E-165	
			Cd9	9.76E-153	
			P2ry12	1.78E-88	
Müller Glia-like (*Rpgr* mutant cells only)	5	-	*Tatdn1*	6.84E-114	N/A
			Rlbp1	1.00E-179	
			Nefl	4.57E-175	
			Glul	3.28E-142	
			mt-Co1	1.89E-121	
			Dbi	5.99E-127	
			Prdx6	5.33E-132	
			mt-Atp8	6.93E-130	
			mt-Nd5	3.13E-112	
			mt-Nd2	1.99E-109	
			mt-Nd1	2.36E-110	
Rod photoreceptor-like (predominantly *Rpgr* mutant cells)	7	-	*Tatdn1*	1.20E-83	N/A
			Nefl	3.96E-140	
			mt-Co1	1.70E-70	
			mt-Nd2	7.36E-70	
			mt-Nd5	2.78E-67	
			mt-Nd1	8.93E-68	
			mt-Atp8	3.06E-53	
			Camk2n1	3.24E-39	
			Rps29	1.46E-53	
			mt-Cytb	2.94E-59	
			Rpl37	3.41E-27	

			Rpl38<br>*Rps28*<br>*Glul*	1.91E-24<br>1.46E-53<br>2.62E-40	

Supplementary Table 1: Marker genes used to identify each cluster as defined by Seurat

Genes listed are the most significantly expressed genes in each cluster compared to all other clusters (ranked by average log_2FC, adjusted p value < 0.0001) and significantly expressed in equivalent clusters in both experiments (if equivalent clusters were present). Differential expression analysis performed by Seurat[4] using non-parametric Wilcoxon rank sum test to determine significance.

Cluster	WT Cell Number	*Rpgr*^{ORFd5} Mut Cell Number	Cluster	WT Cell Number	*Rpgr*^{Ex3d8} Mut Cell Number
0	525	562	0	594	811
1	526	304	1	504	759
2	390	248	2	448	533
3	278	126	3	313	523
4	66	136	4	236	375
5	0	200	5	104	111
6	83	109	6	24	56
7	18	168	7	29	29
8	72	66	8	19	33
9	47	84	9	16	29
10	29	46	10	12	18
11	3	59			
12	5	45			
**Total**	**2042**	**2153**		**2299**	**3277**

Supplementary Table 2: Number of WT and *Rpgr* mutant cells in each cluster as defined by Seurat.

Cluster	% WT rod PR	% *Rpgr*^{ORFd5} rod PR	Cluster	% WT rod PR	% *Rpgr*^{Ex3d8} rod PR
0	30.5	45.3	0	28.4	27.0
1	30.6	24.5	1	24.1	25.3
2	22.7	20.0	2	21.4	17.4
3	16.2	10.2	3	14.9	17.4
			4	11.3	12.5

Supplementary Table 3: Percentage of *Rpgr* mutant and WT rod photoreceptors within each subcluster.

Rpgr^{ORFd5}			*Rpgr*^{Ex3d8}		
**Gene**	**log₂FC**	**pVal_adj**	**Gene**	**log₂FC**	**pVal_adj**
mt-Co1	-1.4893338	3.73E-109	*mt-Nd3*	-0.4191463	3.06E-19
mt-Atp8	-1.3587884	1.50E-118	*Bcl2*	-0.3311563	1.76E-22
mt-Nd2	-1.2949704	4.02E-108	*mt-Cytb*	-0.3260711	5.16E-11
mt-Nd5	-1.2383093	1.88E-92	*mt-Nd4*	-0.3015749	5.72E-10
mt-Nd1	-1.1239659	4.90E-82	*mt-Nd2*	-0.2583465	1.24E-06
mt-Nd4	-1.0520625	3.74E-80	*mt-Nd1*	-0.258555	4.79E-09
mt-Nd3	-1.0380284	4.37E-69			
mt-Nd6	-0.9636443	3.41E-66			
mt-Cytb	-0.9427124	1.04E-68			
mt-Nd4l	-0.8190687	3.48E-49			
Hk2	-0.4155695	2.22E-18			
Ppia	-0.4082531	6.39E-14			
Atp5k	-0.3452921	9.81E-10			
Vdac1	-0.3452309	3.54E-07			

Supplementary Table 4: Genes encoding mitochondrial proteins are up-regulated in *Rpgr* mutant rod photoreceptors. 6 out of 26 genes (23%) significantly up-regulated in *Rpgr*^{Ex3d8} mutant photoreceptors (adjusted p value < 0.0001) and 14 out of 63 genes (22%) significantly up-regulated in *Rpgr*^{ORFd5} mutant photoreceptors are involved in mitochondrial function. Differential expression analysis performed by Seurat[4] using non-parametric Wilcoxon rank sum test to determine significance.

**Gene Symbol**	**-Log p-value**	**Log_2FC**	**Cell Stress Pathways**
Fam57b	2.313307789	2.352181207	Modulation of Ceramide synthesis [5]
Mfap2	2.096796047	2.889847329	Up-regulated by ER stress [6]
Spg20	1.9214212	1.857827801	Removal of damaged lysosomes [7]
Cst3	1.896562643	2.203335257	Oxidative stress; apoptosis regulation [8]
Timm10	1.42582179	1.761368973	Mitochondrial translocase; mitochondrial stress [9]
Cbr3	1.375487739	1.657601449	Target of Nrf2 oxidative stress response [10]
Ak4	2.072904913	-2.24235180	Oxidative stress modulation; cell survival [11]
Pfn1	1.811238023	-1.75451914	Mitochondrial homeostasis [12]
Rab3gap1	1.748810265	-2.07069335	ER-golgi trafficking; ER stress [13]
Grpel1	1.703691058	-1.23502430	Mitochondrial UPR in response to cell stress [14]
Hspe1	1.531379355	-1.54078998	Mitochondrial homeostasis; mitochondrial stress [15]
Pon1	1.476293654	-2.04346807	Protective against oxidative stress [16]
Me2	1.440690924	-1.58867868	Pyruvate metabolism; oxidative stress [17]
Rab2a	1.427479272	-1.20744067	AMPK signalling pathway; ER stress [18]
Sgpp1	1.404529831	-1.92382548	Sphingolipid signalling; Sphingolipid metabolism; ER stress & autophagy [19]
Rtn1	1.387902448	-1.35058868	ER stress; mitochondria- associated apoptosis [20]
Wtap	1.271938595	-1.3143139	ER stress; apoptosis[21]

Txn1	1.260010233	-1.65164640	Deficiency causes oxidative stress [22]
Rprd1a	1.232236679	-1.49484892	Stabilises nuclear Nrf2 [23]

Supplementary Table 5: Differentially expressed proteins in *Rpgr*^{Ex3d8} retina compared to wild type have roles in cell stress pathways.

Genes associated with differentially expressed peptides identified in whole retina mass spectrometry [24] (>1.5 fold enrichment ($log_2FC > 0.6$ or <-0.6) and $p > 0.05$) with functions in cell stress pathways. 6 out of 31 (19%) significantly up-regulated proteins and 13 out of 82 (16%) down-regulated proteins in *Rpgr*^{Ex3d8} retina are associated with cell stress pathways, particularly oxidative and mitochondrial stress.

References:

1. Chen, E. Y. *et al.* Enrichr: interactive and collaborative HTML5 gene list enrichment analysis tool. *BMC Bioinformatics* **14**, 128 (2013).
2. Kuleshov, M. V. *et al.* Enrichr: a comprehensive gene set enrichment analysis web server 2016 update. *Nucleic Acids Res.* **44**, W90-7 (2016).
3. Xie, Z. *et al.* Gene Set Knowledge Discovery with Enrichr. *Curr. Protoc.* **1**, e90 (2021).
4. Stuart, T. et al. Comprehensive Integration of Single-Cell Data. Cell 177, 1888-1902.e21 (2019).
5. Tomasello, D. L. *et al.* 16pdel lipid changes in iPSC-derived neurons and function of FAM57B in lipid metabolism and synaptogenesis. *iScience* **25**, 103551 (2022).
6. Le Thomas, A. *et al.* Decoding non-canonical mRNA decay by the endoplasmic-reticulum stress sensor IRE1α. *Nat. Commun.* **12**, 7310 (2021).
7. Gahlot, P. *et al.* Lysosomal damage sensing and lysophagy initiation by SPG20-ITCH. *Mol. Cell* **84**, 1556-1569.e10 (2024).
8. Nishio, C., Yoshida, K., Nishiyama, K., Hatanaka, H. & Yamada, M. Involvement of cystatin C in oxidative stress-induced apoptosis of cultured rat CNS neurons. *Brain Res.* **873**, 252–262 (2000).
9. Sokol, A. M., Sztolsztener, M. E., Wasilewski, M., Heinz, E. & Chacinska, A. Mitochondrial protein translocases for survival and wellbeing. *FEBS Lett.* **588**, 2484–2495 (2014).
10. Malátková, P., Ebert, B., Wsól, V. & Maser, E. Expression of human carbonyl reductase 3 (CBR3; SDR21C2) is inducible by pro-inflammatory stimuli. *Biochem. Biophys. Res. Commun.* **420**, 368–373 (2012).
11. Liu, R. *et al.* Enzymatically inactive adenylate kinase 4 interacts with mitochondrial ADP/ATP translocase. *Int. J. Biochem. Cell Biol.* **41**, 1371–1380 (2009).
12. Read, T.-A. *et al.* The actin binding protein profilin 1 is critical for mitochondria function. *BioRxiv* (2023) doi:10.1101/2023.08.07.552354.
13. Ghate, P. S. *et al.* The Warburg micro syndrome protein RAB3GAP1 modulates neuronal morphogenesis and interacts with axon elongation end ER-Golgi trafficking factors. *Neurobiol. Dis.* **184**, 106215 (2023).
14. Ma, C. *et al.* GrpEL1 regulates mitochondrial unfolded protein response after experimental subarachnoid hemorrhage in vivo and in vitro. *Brain Res. Bull.* **181**, 97–108 (2022).
15. Yeung, N., Murata, D., Iijima, M. & Sesaki, H. Role of human HSPE1 for OPA1 processing independent of HSPD1. *iScience* **26**, 106067 (2023).
16. Ng, C. J. *et al.* The paraoxonase gene family and atherosclerosis. *Free Radic. Biol. Med.* **38**, 153–163 (2005).

17. Chen, K.-C. *et al.* Targeting human mitochondrial NAD(P)+-dependent malic enzyme (ME2) impairs energy metabolism and redox state and exhibits antileukemic activity in acute myeloid leukemia. *Cell Oncol (Dordr)* **46**, 1301–1316 (2023).
18. Sugawara, T., Kano, F. & Murata, M. Rab2A is a pivotal switch protein that promotes either secretion or ER-associated degradation of (pro)insulin in insulin-secreting cells. *Sci. Rep.* **4**, 6952 (2014).
19. Lépine, S. *et al.* Sphingosine-1-phosphate phosphohydrolase-1 regulates ER stress-induced autophagy. *Cell Death Differ.* **18**, 350–361 (2011).
20. Gong, L. *et al.* RTN1-C mediates cerebral ischemia/reperfusion injury via ER stress and mitochondria-associated apoptosis pathways. *Cell Death Dis.* **8**, e3080 (2017).
21. Wang, J. *et al.* WTAP promotes myocardial ischemia/reperfusion injury by increasing endoplasmic reticulum stress via regulating m6A modification of ATF4 mRNA. *Aging (Albany NY)* **13**, 11135–11149 (2021).
22. Ohmori, I. *et al.* Thioredoxin deficiency increases oxidative stress and causes bilateral symmetrical degeneration in rat midbrain. *Neurobiol. Dis.* **175**, 105921 (2022).
23. Feng, X. *et al.* RPRD1A stabilizes NRF2 and aggravates HCC progression through competing with p62 for TRIM21 binding. *Cell Death Dis.* **13**, 6 (2021).
24. Megaw, R. et al. Ciliary tip actin dynamics regulate photoreceptor outer segment integrity. Nat. Commun. 15, 4316 (2024).
