# Supplementary material for: Autophagy disruption and mitochondrial stress precede photoreceptor necroptosis in multiple mouse models of inherited retinal disorders
Source: Nat Commun. 2025 Apr 29;16:4024. doi: 10.1038/s41467-025-59165-8 (PMC12041483; doi:10.1038/s41467-025-59165-8)
Supplement: Supplementary file 2 — Reporting Summary [file 41467_2025_59165_MOESM2_ESM.pdf]

nature portfolio

Corresponding author(s): Roly Megaw

Last updated by author(s): Apr 1, 2025

Reporting Summary

Nature Portfolio wishes to improve the reproducibility of the work that we publish. This form provides structure for consistency and transparency in reporting. For further information on Nature Portfolio policies, see our Editorial Policies and the Editorial Policy Checklist.

Statistics

For all statistical analyses, confirm that the following items are present in the figure legend, table legend, main text, or Methods section.

n/a	Confirmed	
☐	☒	The exact sample size (*n*) for each experimental group/condition, given as a discrete number and unit of measurement
☐	☒	A statement on whether measurements were taken from distinct samples or whether the same sample was measured repeatedly
☐	☒	The statistical test(s) used AND whether they are one- or two-sided<br>*Only common tests should be described solely by name; describe more complex techniques in the Methods section.*
☒	☐	A description of all covariates tested
☒	☐	A description of any assumptions or corrections, such as tests of normality and adjustment for multiple comparisons
☐	☒	A full description of the statistical parameters including central tendency (e.g. means) or other basic estimates (e.g. regression coefficient) AND variation (e.g. standard deviation) or associated estimates of uncertainty (e.g. confidence intervals)
☒	☐	For null hypothesis testing, the test statistic (e.g. *F*, *t*, *r*) with confidence intervals, effect sizes, degrees of freedom and *P* value noted<br>*Give P values as exact values whenever suitable.*
☒	☐	For Bayesian analysis, information on the choice of priors and Markov chain Monte Carlo settings
☒	☐	For hierarchical and complex designs, identification of the appropriate level for tests and full reporting of outcomes
☒	☐	Estimates of effect sizes (e.g. Cohen's *d*, Pearson's *r*), indicating how they were calculated

Our web collection on statistics for biologists contains articles on many of the points above.

Software and code

Policy information about availability of computer code

Data collection	Description of all code used to collect data is described in the relevant sections of the methods with references.
Data analysis	Description of all code used to analyze data is described in the relevant sections of the methods with references. All statistical analyses used are described in the figure legends along with n numbers for biological replicates and P values. Raw data for biological and experimental replicates has been plotted, where possible. Statistical methods used for analysis are mentioned in each figure as explained in Methods.

For manuscripts utilizing custom algorithms or software that are central to the research but not yet described in published literature, software must be made available to editors and reviewers. We strongly encourage code deposition in a community repository (e.g. GitHub). See the Nature Portfolio guidelines for submitting code & software for further information.

Data

Policy information about availability of data

All manuscripts must include a data availability statement. This statement should provide the following information, where applicable:

- Accession codes, unique identifiers, or web links for publicly available datasets
- A description of any restrictions on data availability
- For clinical datasets or third party data, please ensure that the statement adheres to our policy

Provide your data availability statement here.

Research involving human participants, their data, or biological material

Policy information about studies with human participants or human data. See also policy information about sex, gender (identity/presentation), and sexual orientation and race, ethnicity and racism.

Reporting on sex and gender	N/A
Reporting on race, ethnicity, or other socially relevant groupings	N/A
Population characteristics	N/A
Recruitment	N/A
Ethics oversight	N/A

Note that full information on the approval of the study protocol must also be provided in the manuscript.

Field-specific reporting

Please select the one below that is the best fit for your research. If you are not sure, read the appropriate sections before making your selection.

[x] Life sciences [ ] Behavioural & social sciences [ ] Ecological, evolutionary & environmental sciences

For a reference copy of the document with all sections, see nature.com/documents/nr-reporting-summary-flat.pdf

Life sciences study design

All studies must disclose on these points even when the disclosure is negative.

Sample size	Within the legends of each figure, we described sample size.
Data exclusions	All raw data is plotted within graphs- there was no exclusion of individual samples or data points. All data sets of scRNAseq used in this study will be publicly available upon acceptance of this paper.
Replication	Replicate information, including the number of experiments, biological and technical replicates, is provided in the figure legends, Methods and Source Data file. We use biological replicate to indicate an independent animal. We refer to repeat measurements of the same animal as a technical replicate. The number of data-points per condition is indicated in each sample. The means of the technical replicates for each biological replicate are indicated as a separate symbol overlay on each graph.
Randomization	Experimental groups were assigned based on animal genotype. For image analysis, care was taken to ensure images were taken at random by choosing fields of view based on DAPI, or a stain that was not subsequently being analyzed. In most cases, images were analyzed blinded to genotype for image analysis, and if possible, image analysis was automated to reduce bias.
Blinding	Where possible, samples were blinded for all image acquisition and image analysis.

Reporting for specific materials, systems and methods

We require information from authors about some types of materials, experimental systems and methods used in many studies. Here, indicate whether each material, system or method listed is relevant to your study. If you are not sure if a list item applies to your research, read the appropriate section before selecting a response.

Materials & experimental systems

n/a	Involved in the study	
[ ]	[x]	Antibodies
[x]	[ ]	Eukaryotic cell lines
[x]	[ ]	Palaeontology and archaeology
[ ]	[x]	Animals and other organisms
[x]	[ ]	Clinical data
[x]	[ ]	Dual use research of concern
[x]	[ ]	Plants

Methods

n/a	Involved in the study	
[x]	[ ]	ChIP-seq
[x]	[ ]	Flow cytometry
[x]	[ ]	MRI-based neuroimaging

Antibodies

Antibodies used	We have provided all of this data (name, catalogue number, clone number, species, fixation, dilution, distributor) in the Methods

	section.
Validation	All antibodies used in this study have been used as validated on manufacturer's website and are widely used by the field.

Animals and other research organisms

Policy information about studies involving animals; ARRIVE guidelines recommended for reporting animal research, and Sex and Gender in Research

Laboratory animals	All details of strain, genotype, sex and age are detailed in the methods and in figure legends.
Wild animals	No wild animals were used in this study.
Reporting on sex	Male mice were used for all experiments involving Rpgr mutants (since Rpgr is X-linked). A Mix of male and female mice were used for Pde6batrd2 experiments.
Field-collected samples	No field related samples were used in this study.
Ethics oversight	All experiments followed international, national and institutional guidelines for the care and use of animals. Animal experiments were carried out under UK Home Office Project Licenses PPL P1914806F and PP5301383 in facilities at the University of Edinburgh (PEL 719 60/2605) and were approved by the University of Edinburgh animal welfare and ethical review body.

Note that full information on the approval of the study protocol must also be provided in the manuscript.

Plants

Seed stocks	N/A
Novel plant genotypes	N/A
Authentication	N/A
